# Understanding food vulnerability and health literacy in older bereaved men: A qualitative study

**DOI:** 10.1111/hex.12574

**Published:** 2017-05-24

**Authors:** Jill Thompson, Angela Tod, Paul Bissell, Michael Bond

**Affiliations:** ^1^ School of Nursing and Midwifery The University of Sheffield Sheffield UK; ^2^ School of Health and Related Research The University of Sheffield Sheffield UK; ^3^ Human and Health Sciences The University of Huddesfield Harold Wilson Building (HW2) Queensgate Huddersfield HD1 3DH UK

**Keywords:** bereavement, food vulnerability, health literacy, inequalities, older men

## Abstract

**Background:**

Older people are sometimes challenged in maintaining a healthy diet but, because of age and disadvantage, are also more vulnerable to the adverse health consequences of poor nutrition. It has been claimed that older adults have low levels of health literacy regarding food and struggle to discern which foods are healthy from the vast range available in developed counties. However, nutrition and eating behaviour are modifiable risk factors for health in old age and health benefits can accrue from promoting healthy eating later in life. In order to achieve these health benefits, it is necessary to understand more about the capabilities and vulnerabilities of older people in terms of acquiring and maintaining a healthy diet.

**Objective:**

To understand the potential for issues around food vulnerability to arise in that group and to characterize that vulnerability, if present.

**Design:**

Narrative interviews were conducted to collect the data. An interpretative thematic approach to analysis was utilized.

**Participants:**

Twenty older, bereaved men from two communities in the North of England.

**Findings:**

Five overarching themes were identified: financial security, social networks, cooking skills, food and routine and single servings.

**Discussion:**

Our findings suggest that some older men experience cumulative benefit from resources at their disposal, which contributes towards their capabilities to avoid food vulnerability.

## 1 INTRODUCTION

Basic nutrition is essential for health and well‐being and a key public health issue.^1^ Poor diet has been linked to shorter life expectancy in developed countries.^2^, ^3^ For older adults, and other vulnerable groups, healthy diet and good nutrition are even more important. Diet is one of few modifiable risk factors for health in older age.^2^ Without a balanced diet, older people are at increased risk of earlier death, impaired functional ability, difficulties with activities of daily living, falls, avoidable hospital admissions and longer lengths of hospital stays.^4^ In short, in an ageing population, promoting good nutrition for older people can improve both length and quality of life. By avoiding the adverse consequences of a poor diet, the burden of care on health and social care services is also reduced.

Biologically, the ageing process impacts on appetite and eating habits and, as a consequence, nutritional intake. For example, changes to sense of smell and taste can effect appetite and food choice for older people. These changes become more common between 70 and 90 years of age.^4^ However, the relationship between ageing and food should be explored, not just in relation to biological impacts of ageing as described, but also in relation to social and cultural influences, for example changes in living conditions and arrangements, relationships, income and personal beliefs.^5^ Despite its public health importance, we know little about how best to promote good nutritional standards amongst certain vulnerable groups, including older men. A focus on men who are bereaved is justified because maintaining nutritional standards may become a struggle due to the practical and emotional challenges linked to their loss.

### 1.1 Older men, food and eating behaviour

If we consider physiological and socio‐cultural influences on food intake and behaviour, there is an argument that older men may be particularly vulnerable to poor nutrition. However, there is little research on experiences and attitudes to health and food that focuses on men of any age, but especially for older men. Most research has explored women's experiences in this area. “Traditional feminization” of food purchase and cooking roles within households may explain this lack of research in men.^6^, ^7^ In addition, there are assumptions that men rely on women for advice and support about food and health.^8^ Evidence indicates that gender divisions of labour remain in many households in the United Kingdom, with food shopping, preparation and cooking often organized by women.^9^ Similarly, food advertising, health literacy and health promotion messages are heavily gendered.^10^ They target women and play to the traditional gender roles in white Western households. As a result, health promotion messages in relation to food tend not to engage with men, or older men.

Literature exploring food and identity shows associations between food type and gender. For example, red meat and hearty portion sizes are coded as masculine, whereas “feminine foods” reflect lighter options such as fish, fruit and yogurt.^6^, ^11^ Current health promotion and health literacy messages warn against overconsumption of the former whilst promoting the latter. Furthermore, research has highlighted how conventional masculinities of male autonomy and choice can prevent some men from acting on (and in some cases actively resisting) government healthy eating directives and health promotion initiatives.^6^

There is a strong link between older men's awareness of nutrition and health and the food that they consume.^7^ In general, older men living alone have a less than adequate diet, with less variety and a poorer understanding of nutrition than older women living alone.^9^ Furthermore, it has been reported that bereavement often leaves older men ill‐prepared for looking after their own eating and nutritional needs, due to lack of cooking skills and nutritional knowledge.^12^

Food and pleasure is identified as a potential area for exploration in relation to older men and food.^7^ Eating alone removes the social aspect of food.^7^, ^13^ Thus, social isolation can reduce the meaning and pleasure of food, evoking a sense of vulnerability.^7^ Access to transport is also a potential influencing factor in older men's enjoyment of food.^14^ It provides a means to engage in social activities and shared meals. Older men without access to transport have been reported to be at increased risk of social isolation and less likely to engage in social activities involving food.^14^ Widowed men living alone are at particular risk of social isolation. If they are struggling to buy, cook and eat a nutritious diet, this may go unnoticed until it impacts negatively on health and they present to services.^7^

### 1.2 Bereavement and food vulnerability

Bereavement can have a negative impact on the nutritional status for widowed men and women.^15^ Emotional stress, social isolation and loneliness have all been found to negatively impact on an individual's motivation and desire to eat, and eat well, following bereavement.^15^

Given the physiological, social and gendered nature of food related behaviour, summarized here, older bereaved men could be a particularly vulnerable group in terms of diet and nutrition. This is especially true for men from traditional households where their wives controlled food purchase, cooking decisions and preparation.^7^, ^14^ In these circumstances, the onset of their wives’ illness, or death, may mark the first encounter that many older men have with food purchase and cooking.^14^ A small number of studies suggest that some older men who find themselves responsible for cooking later in life relish the challenge. However, other older bereaved men have been found to struggle, lacking necessary skills and knowledge of food purchase and preparation to maintain an adequate and varied diet.^16^, ^17^

This review of the current limited evidence indicates that a range of multidimensional factors may affect how challenged older bereaved men are in maintaining a healthy diet. They can create nutritional needs but can also create barriers to meeting these needs. Other factors relate to information and awareness, food and nutrition skills, social and cultural norms, gender, and wider social determinants of health.

In order to understand how influences, risk and vulnerability play out in relation to food behaviour in older bereaved men, this study aimed to examine their everyday food practices and experiences, and explore their attitudes, behaviour and knowledge concerning food and nutrition. The theoretical concepts used in the study are health literacy and food vulnerability.

### 1.3 Health literacy

Health literacy is a complex term that relates to a range of outcomes of health promotion or education activity.^18^ Traditionally, health literacy focused on functional information provision and communication to promote literacy regarding health‐related materials, for example prescriptions, appointments and medicine labels. However, if we are to promote healthy eating behaviour change, it is necessary to think of health literacy as a more complex endeavour, not just communicating information. Nutbeam^18^, ^19^ proposed a classification of health literacy that incorporated three levels (functional, interactive and critical health literacy), and Velardo^19^ relates these to food and nutrition (Table 1):

**Table 1:** Health literacy and nutrition

Functional nutrition literacy	The ability to obtain food nutrition information and to develop an understanding of nutritional factors that can enhance or inhibit good health
Interactive nutrition literacy	The ability to translate nutrition information into positive dietary choices and having the skills to interpret and evaluate nutrition information and to prepare healthy food
Critical nutrition literacy	Skills associated with identifying seasonal produce, or growing your own food. Considering the wider impact of individual and community food choices

From: Nutbeam^18^, ^19^ and Verlado.^19^

This broader classification prompts not just the transfer of information for individual benefit (functional health literacy). It also facilitates improved capacity to act on knowledge and influence social norms (interactive health literacy). Critical health literacy would seek to improve individual resilience and agency and address social and economic determinants of health to improve capacity, assets and empowerment at a community and individual level.

Existing evidence relating to older widowed men, food and health indicates that some do better than others in maintaining a healthy diet. Information is only one influence. Wider environmental, economic and social determinants of nutritional risks include transport, social connections, income and education. There is a need for more in‐depth qualitative exploration of older bereaved men's eating and food experiences using Nutbeam's^18^ classification of health literacy to frame the inquiry.

### 1.4 Food vulnerability

Vulnerability is a core concept when understanding health, risk and harm. Traditionally, vulnerability has been applied at a population level, in epidemiology and public health, to identify groups and communities at high risk of a health threat or illness. However, work by nursing theorist, Speirs,^20^ revisited the concept. Spiers^20^ considered vulnerability from an emic perspective, exploring an individual's own experiences of health in their day‐to‐day lives. Four primary attributes of emic vulnerability are proposed: integrity, challenge, capacity for action and multidimensionality (Table 2). These attributes provide a framework to apply and understand vulnerability from specific risks. The framework has been successfully applied to other health‐related areas including “energy vulnerability.” 21 It has helped to understand who is vulnerable, why they are vulnerable, and what can be done to reduce vulnerability.

**Table 2:** Food vulnerability

Spiers’ attributes of emic vulnerability	Definitions	Application to food vulnerability
Integrity	“The person's sense of soundness in the various dimensions in his life”	The ability to make healthy food decisions (eg, purchase, preparation and cooking) and maintain a healthy diet and
Challenge	“Vulnerability is experienced when there is a perceived challenge to integrity with a corresponding uncertainty about the ability to respond”	Anything that challenges a person or household's ability to make healthy food decisions (eg, purchase, preparation and cooking) and maintain a healthy diet
Capacity for action	“Capacity for action refers to the individual's perceived ability to withstand, integrate or cope with the challenge”	How an individual or household copes with (and perceives themselves coping with) the challenges to their ability to make healthy food decisions (eg, purchase, preparation and cooking) and maintain a healthy diet
Multidimensionality	“The fact that vulnerability varies from one person to another and from one experience to another”	How food vulnerability is experienced differently by different people in different circumstances

Adapted from Middlemiss & Gillard.^21^

Underpinning the study presented here is an intention to understand and characterize food vulnerability in older, bereaved men. Porter Starr et al.^22^ suggest food vulnerability as evoking a classic image of a home‐bound older person with limited resources and/or medical disabilities that impede their ability to consume an adequate diet. But such images evade the inherent complexities of eating in older age.

Using similar techniques as Middlemas and Gilard,^21^ we apply Spiers’^20^ attributes of emic vulnerability to a food vulnerability context. Drawing on this perspective, one could hypothesize that being health literate, from a functional, interactive and critical perspective, may reduce food vulnerability.

## 2 METHODS

Qualitative interviews, drawing on narrative approaches, were used. Narrative interviews are largely unstructured and are appropriate when exploring sensitive issues and when researchers wish to “minimize” their own agenda and limit their influence on the data that are collected.^23^ Given the focus of the study, this methodological approach would enable participants to construct their own stories, or narratives, about food and to discuss their wife/partner, and their bereavement, as and when they chose to. Each participant constructed differing accounts of food purchase and cooking over their life course, highlighting important indexical statements.^24^

Whilst the narrative approach gives a certain degree of control over to the participant to direct the process, as with all qualitative research, we must also recognize and examine our own involvement in shaping the data that are collected and how it is analysed.^24^

### 2.1 Sampling

Interviews were undertaken with 20 older bereaved men between September 2013 and January 2014. Participants were identified and initially contacted through the Yorkshire Cohort Study.^25^, ^26^ The YCS is a population cohort that aims to track the health of participants over time. With consent of participants, the YCS also serves as a recruitment facility for other research.

We sent out a screening questionnaire to every man aged 65 and over who was registered on the YCS (n=1805). From this, we received 744 replies, of which 85 were widowed. Each of these individuals was sent an invitation to take part in an interview and we recruited the first 20 men who responded and whom we were able to arrange an interview within the time limitations of our study.

### 2.2 Sample

Participants were aged between 65 and 90 years with an average age of 80. All men had been bereaved at least 6 months, with the longest length of bereavement 30 years and the average 1‐5 years.

All participants in our study were white British, living in one of two communities in the North of England. Previous occupations included the police force, teaching, lecturing, management and business ownership.

### 2.3 Interviews

Face‐to‐face interviews were arranged at a date, time and place convenient to the participants. The interviews were conducted by JT and MB and lasted 45‐90 minutes. Interviews were audio recorded with the participant's consent and handwritten written notes made throughout the process.

In keeping with the methodological approach, the interview guide was flexible enough to allow individual narratives to emerge. Topics covered changing experiences of food purchase, preparation and cooking throughout the life course; significant events that may have impacted on the participant's food preferences, consumption and nutrition throughout their life, the impact of their wife/partners illness (if applicable) and death on the participants’ food preferences, consumption and nutrition; and any services and support (formal or informal) that the participants utilized with regard to food and nutrition.

### 2.4 Data analysis

Interviews were digitally recorded, transcribed verbatim and anonymized. Transcripts were shared with participants for comment. An interpretative thematic approach to analysis was used.^27^ This was a reflexive and iterative process, exploring participants’ accounts in their entirety before undertaking initial open coding followed by selective and more detailed coding. Links, comparisons and relationships were explored between themes, across participants and with the wider literature.

### 2.5 Ethics

The study received the required NHS research ethics and governance approval. Ethics approval was granted by Derbyshire NHS Research Ethics Committee.

## 3 FINDINGS

The findings reveal a number of influences on health literacy and vulnerability in relation to food and health for older bereaved men. We present the findings here as five overarching themes: financial security, social networks, cooking skills, food and routine, and single servings and consider these findings in the context of health literacy and food vulnerability.

### 3.1 Financial security

The participants within our study identified themselves as financially solvent and many owned their home outright. No participants suggested that they were struggling financially or that finance restricted their ability to purchase adequate food. Some men identified themselves as better off in retirement than when working, whilst others felt that their lifestyle was modest but not extravagant:

My income's better now I've retired than it was before. It may sound strange but pensions started coming in from all over the place! (Alex)

Furthermore, the majority of men in our study had access to transport or were physically able to access shops and social groups within easy walking distance, reducing their social isolation. Thus, in terms of the environmental and economic means to purchase a nutritionally varied diet, the participants in our study appeared capable and secure and in terms of food vulnerability, the challenges were reduced.

### 3.2 Social networks

The majority of our participants could be regarded as well networked socially, with family, friends and social activities featuring heavily in their accounts of food and eating. Three‐quarters of the participants had children whom they saw on a regular basis. They described meals with children as part of their weekly or monthly routines. Two of the participants had an adult child living with them—one of whom was acting as their father's full‐time caregiver.

Many of the participants spoke fondly of their community, neighbourhood and/or immediate neighbours. In relation to food, a number of participants spoke of their neighbours helping with food purchase and in some cases, close neighbours would come together to share a meal or drink. This contributed to participants’ sense of integrity, reducing the potential for food vulnerability. Strong communities were identified as support networks following participants’ bereavement:

I can't think of anybody better than the local community and my friends, they are the people who have been my salvation. The fact that they can, you know, you can be yourself with them, you can discuss things with them and I mean there are far more widowers but they've gone through the same situation as I've gone through and we can talk normally. (Christopher)

Over half of the participants identified established social routines based around, or involving food. This included routine visits to, or from, family or friends and membership of community and social groups. Participants within our study were members of numerous community and social groups including the church, sports groups, Probus, wine guilds and dancing groups.

For example, Alex was part of a friendship network with a regular poker night:

I have a poker night here where some friends come round and we have a game of poker and I always prepare something for them to eat before that. The last two times I did a couple of curries out of the Rick Stein book.

In this example, food preparation was undertaken for a larger number of people and thus regarded as worthy of the effort required. This was also referred to by a number of participants, who felt that whilst they had the capability to cook for themselves, the social context provided the motivation, or capacity of action, to undertake these activities:

I'm quite happy to cook for myself providing I've got it, somebody to do it for, whether it's sons or friends or relations. (Douglas)

Another participant spoke of food as a motivating factor for his engagement with social activities:

…if there is a party or a do, I will go to it. Mainly to get fed. (Neil)

Clearly, food featured widely in participants’ accounts of their social lives, with many of our participants presenting themselves as part of strong social networks. Despite this, one participant (David) could be described as socially isolated, with only a single family member visiting him on a fortnightly basis. Other than this visit, David did not attend any social activities, did not consider himself to have any friends or close social networks and rarely ate out. Food, for David, had lost its social meaning:

…I usually try and cook something, but it is not always easy to do it because there is a lack of incentive to do it. You don't feel as if you can be bothered to do it for yourself

The process of eating alone when bereaved was found to reduce motivation in food and provided a challenge in terms of an individual's potential for food vulnerability, reducing their capacity for action. Furthermore, the changing place of food consumption following bereavement was also raised by a small number of participants. For example, food consumed at the dining table when their wife or partner was alive, and eating at a table with their young families, was contrasted against eating in front of the television, or off a tray, as a single person:

Doris used to insist we ate at the table, even when there was just two of us. Now I eat, I rarely set a table. Although last weekend my son came up with, they have just had a new baby and we ate as mum would have had it, at the table. But now I normally eat off a tray. (Robert)

These experiences highlight the importance of ceremony in food and eating. It was often the case that participants’ wives/partners had been central figures in maintaining the ceremony of eating around a dining table. This was rarely maintained when eating alone.

### 3.3 Cooking skills

With few exceptions, the participants described fairly traditional gendered divisions of labour within their households. For the majority of men, their wife was the person responsible for food purchase, preparation and cooking. In such cases, the wife's illness was usually the time when our participants began to get involved with food, developing interactive health literacy with varying degrees of interest. Some men spoke positively, enjoying new challenges and identifying their capacity for action, in developing new skills.

…I never got seriously into cooking until [name removed] got ill, because she had lung cancer and also had tertiary tumours on her spine, so it made it very difficult to move around. So I was her carer for the two years that she had cancer and I did all the cooking then and she was very good. She helped me, she put up with some major disappointments but yeah it was good fun. I really enjoyed it. I really enjoyed getting into cooking at that time. (Alex)

On the other hand, some of our participants had developed an interest in food after their wife's death and spoke enthusiastically about their continued efforts to develop their skills and abilities and interactive health literacy.

…my daughter said ‘Dad can you ice the wedding cake? So in actual fact I went to night school at the age of 65 and did a City and Guilds course. I didn't take the exam but I was in the City and Guilds class for cake decoration’. (Christopher)

As part of the enjoyment of food, a small number of our participants identified themselves as keen gardeners and incorporated food that they grew themselves into their daily diet, demonstrating critical health literacy skills:

…I have got a greenhouse. I grow tomatoes in there and squash. The squash I mainly give to my daughter‐in‐law. She makes soup with the squash. I have got a load of blackberries and gooseberries in the freezer actually because I had a good crop…I use those for making myself crumbles (Arthur)

Such examples are illustrative of a capacity for action in terms of reducing potential food vulnerability. Furthermore, a few of our participants displayed awareness of key health messages including “5‐a‐day,” limiting red meat and sodium intake, thus demonstrating their functional and interactive health literacy.

Others expressed less interest in food, or developing interactive health literacy, and struggled to change entrenched behaviours and attitudes. Some attitudes clearly related to gender identity:

Well I tell you I am old school. I believe women do the cooking and washing, men go to work…Well I can't cook and for me to be a cook I have got to be interested in it…It's just my way, I am not bothered about learning to cook. So I can cook, make things, not much. I couldn't cook a Sunday roast or owt like that. I can do chips and I do next to nowt. What I do, I eat out. (Richard)

Despite some participants expressing a disinterest in cooking, only a minority referred to convenience foods in their daily food practices. The majority of the men in our study could be described as relatively self‐sufficient in terms of food purchase, preparation and cooking.

### 3.4 Food and routine

Routine emerged as important in some men's accounts of how they maintained capacity for action with regard to food purchase and eating behaviour. Keeping things simple helped. For example, one participant described how he bulk bought mince beef at the beginning of the week and consumed it daily, perhaps varying the vegetables that he added to it. Another participant took a similar approach by following the same daily food routine:

Typical of what I have had today is typical of what I generally have. So it is veg. I put some veg in. Cabbage and what is the other one called, broccoli. I use broccoli quite honestly because it is easy to cook. It cooks quicker. Cabbage takes longer to soften it. So to me that is all saving money really at the same time. So it doesn't take long really. I put some broccoli in, chop a carrot up, couple of small onions, chop them up…Oh and what I do on top of that for an extra bit of protein, I get the oil fish. Pilchards generally in a tin admittedly but not quite as good as fresh fish but it is a lot cheaper to be honest at the end of the day… But essentially I repeat that twice a day. So you can tell it is fairly basic. (Peter)

Food consumption in this sense appears to be more akin to survival than enjoyment. Speed, efficiency and money saving were favoured over potential food enjoyment and variety. Furthermore, the lack of variety in these men's daily eating practices raises potential nutritional concerns.

Whilst simplicity and routine helped some participants in their daily food practices, it was not true for all. One participant highlighted his active resistance towards set routines regarding this as a marker of old age:

I try to avoid having the same thing each day…Well because I don't want to get set in a routine, you know which is easy to do as you get older. (Arthur)

In addition to routines based on food types, the majority of participants in our study discussed eating routines that were based on a social context. Dining out at restaurants or eating at family or friends’ houses were commonly identified routines as previously discussed.

### 3.5 Single servings

A commonly expressed vulnerability after the participant's bereavement related to the challenge of shopping and cooking for one. They highlighted how supermarkets rarely catered for single people, offering incentives based on bulk buying which would appeal to larger families:

It is difficult to buy for one as well. What annoys me is buy one get one free. You know if a loaf is buy one get one free and then I finish up feeding a loaf to the birds, which is well good for the birds…Shops are not geared to people who are on their own. And I feel a bit guilty if I buy things in small quantities. You know go up to the till and you have got two apples and two bananas and one orange. It is a bit embarrassing buying in small quantities when the lady in front with two kids has got a trolley full of you know and I am there with my little basket (Robert)

Shopping for one person presented as a challenge for individuals in terms of their potential for food vulnerability. Robert spoke of his embarrassment when buying small quantities. He felt conspicuous in food and shopping environments that targeted families rather than the individual. Comparing himself to the larger families did not enhance his self‐esteem and may have reinforced his personal sense of social isolation.

Building on this, a few participants described the effort required to cook for themselves, with many suggesting that it was not worth the effort or outcome:

I usually try and cook something but it is not always easy to do it because there is a lack of incentive to do it. And you don't feel as if you can be bothered to do it for yourself. It is too much effort even for something quite trivial really. (David)

In these accounts, food purchase, preparation and cooking were regarded as activities linked to family life, not the domain of the single person. A particular food vulnerability was identified for those older bereaved men who do not regularly engage in social networks and activities in which food is involved. The data suggest that enjoyment from eating is not about the food itself. Rather, it is the social context of food that enables individuals to engage in aspects of critical health literacy and build their resilience through their sense of belonging.

## 4 DISCUSSION

In this study, we explored older bereaved men's accounts of daily food practices in terms of their food purchases, preparation, cooking and consumption patterns. Based on existing literature, we had expected to identify food vulnerability amongst our participants, attributable to social isolation or a lack of health literacy skills and knowledge to maintain adequate nutrition. Consistent with this expectation, the men in our study highlighted the importance of the social context of food. When social context was removed, men's capacity of action was reduced as eating became a necessity rather than a pleasure. Thus, social isolation was found to increase the potential for food vulnerability. These findings resonate with those from other studies that identify food and meals as social events.^7^

Taking part in social activities where food would be consumed provided the men in our study with a sense of mutuality and support, thus reducing their potential for experiencing food vulnerability. Building on this, availability of food that catered for the individual and the social process of going to a supermarket as an older bereaved man challenged their individual personal emotional integrity.

However, the sample of men in our study were relatively privileged, in terms of the communities in which they resided, their home ownership and financial status. It is important to acknowledge this as a potential limitation to our study. Furthermore, all of our participants were of white British ethnic origin and retired from professional and managerial roles, potentially implying a level of health literacy. Their socio‐economic backgrounds undoubtedly offered protections against the food vulnerabilities we had expected to see. When exploring gendered patterns of food behaviour, social class has been identified as a mediating factor, with research suggesting that men from higher social classes display greater interactive health literacy, taking more of an active interest in healthy eating and cooking, than men from lower social classes.^11^, ^28^ Indeed, the men in our study were generally retired from professional and managerial roles. They had the financial means to purchase a nutritionally varied diet, and often had access to their own or public transport and local facilities. The findings indicate that financial solvency, and the freedom to make choices that was associated with this, were important factors in increasing men's integrity and thus reducing the potential for food vulnerability.

Despite some participants identifying challenges in terms of food purchase and the required efforts to cook for one, many recounted positive stories of food and eating. They displayed clear interactive and critical health literacy skills, with strong social networks identified, contributing towards their capacity for action and reducing their potential for food vulnerability. We were able to generate insight into how bereaved men from a relatively advantaged population were able to overcome some obstacles and barriers to food, highlighted by bereavement.

Certainly in terms of their capacity for action, the majority of individuals in our study identified capabilities for food purchase and preparation. Many had begun to develop these skills during their wives’ illness. Most of those interviewed portrayed later life and widowhood as a time in which they continue to actively participate in society. Indeed, for some, bereavement appeared to have incentivized their engagement in a range of activities based around food consumption that continued their productivity in older age.

Participants in our study experienced the cumulative benefit from the resources that they had at their disposal and these contributed towards their capabilities to avoid food vulnerability. One could theorize that that those from more deprived populations would not have access to such benefits and therefore would be at risk of food vulnerability, and we would suggest that future studies should seek to explore this in more depth.

In the past, the public health response to food vulnerability has been to promote healthy food intake through community cookery classes and improving health literacy.^29^ However, our findings indicate that food vulnerability is a complex issue and a more integrated approach that takes into consideration the financial, transport and social network issues needs to be explored. An important area for public health and future research is how these issues are tackled in populations that do not experience the same cumulative benefits as the men in our study did and thus may be more at risk of food vulnerability.
